# Whole-body vibration transmission during resistance vibration exercise

**DOI:** 10.3389/fspor.2025.1573571

**Published:** 2025-05-06

**Authors:** Riccardo G. Sorrentino, Nina Verdel, Matej Supej, Ursa Ciuha, Igor B. Mekjavic

**Affiliations:** ^1^ Department of Automatics, Biocybernetics and Robotics, Jozef Stefan Institute, Ljubljana, Slovenia; ^2^ Jozef Stefan International Postgraduate School, Ljubljana, Slovenia; ^3^ Faculty of Physical Education, University of Ljubljana, Ljubljana, Slovenia

**Keywords:** vibration transmission, short arm human centrifuge, artificial gravity, vibration exercise, resistance exercise

## Abstract

**Introduction:**

Resistive exercise combined with whole-body vibration (WBV) and short-arm human centrifugation is being considered as a countermeasure to mitigate muscle and bone loss in astronauts during prolonged space missions. WBV may provide exercise benefits or adverse effects on organs and the lower back. These effects may result from vibration transmitted throughout the body. The objective of this study was to compare vibration transmission (VT) from the ground to the body during resistance vibration exercise (RVE) comprising squat and calf raise performed on a rotational vibration plate. Specifically, we compared VT during RVE in the upright position (URVE) and on a short-arm human centrifuge (artificial gravity, AG) establishing a similar ground reaction force. The latter (AGRVE) is considered a potential countermeasure for microgravity-induced musculoskeletal deconditioning.

**Methods:**

Fifteen healthy males participated. They were assigned to two groups: one (*n* = 8) performed URVE at 20% of 1 repetition maximum (RM) squat; the other (*n* = 7) performed horizontal RVE during AG exposure (AGRVE), with a matching ground reaction force. Both groups were exposed to vibration at 20 Hz and 3–4 mm displacement (RMS value: 4 ± 0.14 g). VT was recorded during two sets of squats and calf raises. Three accelerometers recorded VT at: (i) the platform surface at the feet, (ii) lower back (L5), and (iii) forehead.

**Results:**

In both conditions, the lower limbs attenuated vibration transmission to the lower back (*p* < 0.0001). During AGRVE, both VT and pelvis octave-band RMS values were lower compared to URVE in both squat (*p* = 0.008 and *p* = 0.01) and calf raise (*p* = 0.007 and *p* = 0.01), suggesting potentially greater safety for the lower back.

**Conclusion:**

During RVE, whether in URVE or AGRVE, lower limbs effectively attenuated vibrations, resulting in negligible pelvic exposure. AGRVE may represent a safer alternative to URVE due to reduced transmission to the lower back and adjacent sensitive regions.

## Introduction

Whole-body vibration has gained attention due to its potential benefits in muscle activation, bone density improvement (1–4), and neuromuscular conditioning (5–8). In particular, previous research has demonstrated how WBV combined with resistance exercise promotes enhanced musculoskeletal performance and mitigates muscle and bone loss during bed rest (1, 2, 9). Nonetheless, the benefits of resistance vibration exercise remain equivocal, particularly the effect on muscle structure and function, with some studies reporting no additional value over traditional exercise (10–13).

Vibration transmission during vibration training is an important issue, due to the fact that vibrations can cause injuries or harm sensitive areas such as the lower trunk. Vibrations reaching this zone can cause discomfort, nausea, dizziness sensation and motion sickness, along with chronic back pain, if vibrations are experienced consistently (14–17). There is evidence of decreased vibration transmission during exercise or the influence of body posture to dampen vibrations and highly reducing the amount of vibrations reaching the spine (18–22). Several authors proposed the implementation of WBV as a countermeasure for musculoskeletal deconditioning due to space exploration (16, 23, 24), however, during the development of any exercise strategy that incorporates whole-body vibration, the duration of exposure and root mean squared values (RMS), should be within the guidelines recommended by the International Organization for Standardization (ISO 2631-1:1997) and European vibration directive (Directive 2002/44/EC). Current guidelines do not differentiate between occupational and recreational vibration exposure. It is therefore essential that the magnitude of the vibration transmitted from the vibration plate to other regions of the body be determined. This allows a better appreciation of any potential vibration-associated maladies.

The European Space Agency is currently investigating two potential countermeasures: artificial gravity generated via short-arm human centrifuges (SAHC) and whole-body vibration (WBV). This interest arises from the projected conditions of a future mission to Mars, during which astronauts will be exposed to reduced gravitational forces for approximately three years—comprising sustained weightlessness during the return transit and exposure to only 38% of Earth's gravity while on the Martian surface (25). The adaptation of physiological systems to microgravity has been extensively investigated in astronauts by comparing post-mission status of physiological systems with that observed before the mission (25–28). Exposure to microgravity and inactivity/unloading induced by the experimental bed rest, an analog for ground-based studies of the effect of microgravity on physiological systems, significantly impacts the musculoskeletal system by causing the loss of muscle (29) and bone mass (30) resulting in decreased astronauts' performance (31–34). Maintenance of muscle and skeletal mass during space missions is therefore of paramount importance, as reduced performance could increase the risk of injuries and potentially jeopardize mission success. Not surprisingly, a major initiative in space life sciences is the development of effective exercise countermeasures (35, 36).

Short-arm human centrifugation establishes an artificial gravity (AG) vector in the head to foot direction which can mimic gravitational load or even hypergravity (37, 38). This gravitational vector is linear but non-uniform, progressively increasing in magnitude along the length of the nacelle. As a result, participants experience lower loading forces at the shoulder level and progressively greater loads at the feet as presented in literature (23). New generation centrifuges allow users to perform either power (39, 40) and/or resistance exercise (41, 42). The efficacy of horizontal resistance vibration exercise (RVE) conducted with artificial gravity (AGRVE) is the focus of the current ESA BRAVE (**B**ed **R**est **A**rtificial gravity and **V**ibration **E**xercise) project. It is not known whether the unique load distribution during AG exercise would affect vibration behaviour.

Given the unique distribution of gravitational load during centrifugation and the absence of existing research on vibration behaviour in this emerging exercise modality (AGRVE), the present study aimed to investigate whole-body vibration transmission and the magnitude of root mean square (RMS) vibration values during resistance vibration exercise performed on a short-arm human centrifuge (AGRVE) and on an upright exercise device (URVE) under identical vibration frequency and amplitude.

## Methods

### Ethics approval

The study protocol involved human participants and was approved by the University of Ljubljana, Faculty of Sports' Committee for Ethical issues in the field of sport (Reference number: 033-10/2023-2). All participants provided their written informed consent to participate in the study, which was performed according to the guidelines of the Declaration of Helsinki.

### Participants

Recreationally active male participants with prior knowledge of resistance training (*n* = 15) were recruited to take part in the study via online advertisement. Inclusion criteria included regular exercise and familiarity with resistance exercise, specifically squats and calf raises, while the exclusion criteria included the presence of musculoskeletal injuries and low tolerance to motion sickness. Due to the technical constraints of the short-arm human centrifuge (SAHC), individuals weighing over 95 kg or taller than 190 cm were not eligible for inclusion. All participants attended the laboratory on three separate occasions. During the initial visit, participants' ability to perform squat and calf raise exercises was assessed in accordance with established literature guidelines, specifically, achieving a minimum knee flexion angle of 90° during squats and full ankle extension during calf raises. Additionally, the experimental protocol was thoroughly explained to each participant. All individuals demonstrated correct execution of the exercises and met the required performance criteria. During the second visit, anthropometric measurements were recorded, and participants completed an 8 Repetition Maximum (RM) squat test, which was used to calculate the exercise load. The third visit was dedicated to the execution of the experimental protocol.

### Materials

**Figure 1:**
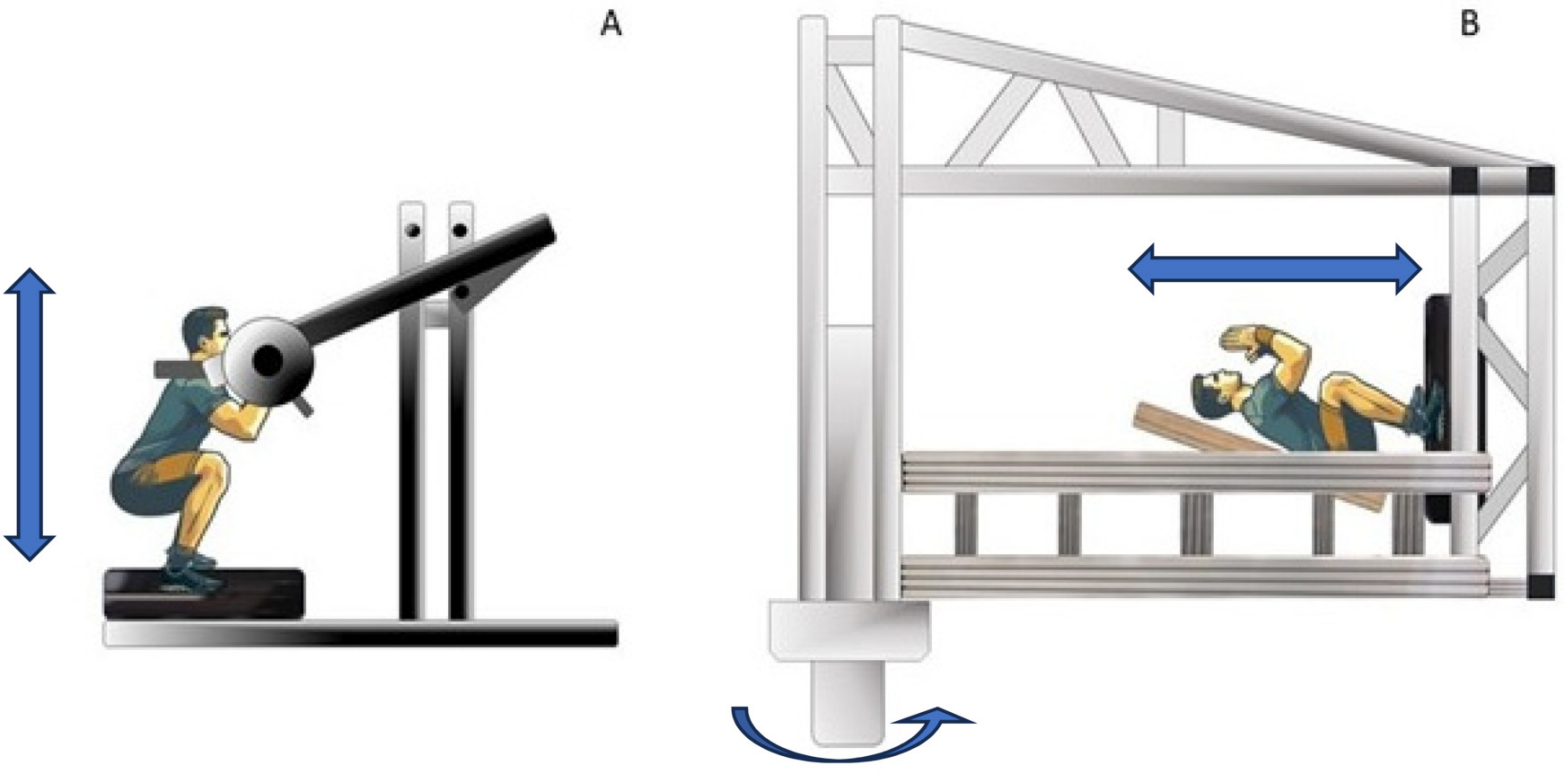
The left panel **(A)** depicts the upright resistance vibration exercise (URVE) conducted with a pendulum device, with the participant standing on the vibration platform. The right panel **(B)** depicts a subject conducting resistance vibration exercise while exposed to artificial gravity (AGRVE) on the Short Arm Human Centrifuge (SAHC). The feet are positioned on a vibration platform located at the end of the nacelle.

Vibration transmission was recorded with two triaxial and one single axis accelerometers (Dytran Instruments, Inc., Chatsworth, USA). These accelerometers were connected to a Dewesoft (Dewesoft d.o.o., Trbovlje, Slovenia) model DEWE-43A data acquisition system. The single-axis accelerometer was calibrated by the manufacturer using known vibration levels applied via a precision shaker, with the accelerometer's output measured through a data acquisition system. The triaxial accelerometers were calibrated by the authors using gravity as a reference, rotating the sensors periodically, thus changing its position in relation to gravity, to measure both + g and -g values. Additionally, the zero-g value was verified by positioning the sensor perpendicularly. Vibrations were generated by a rotational vibration platform (Galileo Space Pro, Novotec, Pforzheim, Germany) specifically manufactured to be used on the SAHC. For this study two platforms were used, one mounted on the SAHC and one placed below the upright exercise device as shown in Figure 1. The rotational vibration platform used in this study had a frequency range of 5–35 Hz, a displacement range of 0.5–5.5 mm amplitude and a maximum acceleration of 27 g.

### Experimental procedure

Participants were randomly assigned to two groups. One group (*n* = 8; age: 27 ± 5 years, bodyweight: 77.75 ± 7.81 kg, height: 182 ± 3.84 cm) performed upright resistance vibration exercise (URVE), and the other group (*n* = 7; age: 22 ± 2 years, bodyweight: 76.4 ± 7.5 kg, height 179 ± 2 cm) conducted RVE on the SAHC, and thus in the presence of artificial gravity centrifugation (AGRVE).

Each participant performed an 8-repetition maximum (8 RM) squat test, and the outcome was used to determine the individualized load intensity (20% of 1 RM). Each test was supervised by two strength and conditioning specialists. Participants started the exercise with a warm up, followed by 2 sets of squats using the Olympic barbells (20 kg, 20.5 mm diameter) and additional 2 sets with a load of half of their bodyweight (i.e., 40 kg including the bar for an 80 kg body mass participant) separated by 1-minute rest between sets. The 8-repetition maximum (8RM) assessment was conducted using a stepwise incremental loading protocol. Load increments were initially set at 10% of the previous load; however, based on participant feedback, increments were adjusted to 5% when the prescribed increase was deemed excessive. The test was terminated when the participant was unable to complete the required number of repetitions or could no longer maintain proper and safe exercise technique. During the calf raise exercise, repetitions were performed with a load equivalent to 1.5 times the participant's BW. The 8-RM results were used to indirectly determined the 1-RM which was 101 ± 13 kg for the URVE group and 96 ± 20 kg for the AGRVE group. The individualized load intensities for squats and calf raises were selected to elicit a moderate level of fatigue typically observed in resistance training, while avoiding excessive fatigue that could compromise movement technique. Additionally, these loads were chosen to strike a balance between simulating realistic exercise conditions for astronaut training and ensuring the reliability of vibration measurements. The vibration plate could provide oscillations between 5 and 35 Hz. In this study the frequency of 20 Hz was chosen. The amplitude of the vibration at the position of the feet on the vibration plate was 3.5 mm. The vibratory load was selected by identifying an intensity, which was sufficiently robust for participants while remaining tolerable during dynamic exercises (24) and because it was observed that during horizontal exercise, higher frequencies coupled with greater amplitudes can cause the feet to lift off the platform, resulting in a loss of continuous contact, thereby making the execution of the exercise difficult and not tolerable. In both groups the exercise was paced with a metronome: 3 s for the down phase and 3 s for the up phase. For the calf raise exercise, participants had to maintain an isometric contraction of 2 s at maximal plantar flexion.

### AGRVE group

The AGRVE trials were conducted on the SAHC (Redwire, Antwerp, Belgium) in the Gravitational Physiology Laboratory of the PlanHab facility in Slovenia (Rateče-Planica, Slovenia). During the first visit, participants were familiarized with the protocol on the short arm human centrifuge. On the third visit, upon arrival at the laboratory, participants changed into gym attire and were fitted with a harness, which was secured to the sides of the cradle system on the SAHC. One integrated electronic piezoelectric single axis accelerometer (sensitivity: 200 mV/g, range: 50 g, mass: 13 g) (Dytran Instruments Inc. Chatsworth, CA, USA) was attached on the vibration platform, next to the participant's foot, at the level of the toes. Two triaxial accelerometers (sensitivity: 100 mV/g, range: 50 g, mass 4.3 g) (Dytran Instruments Inc. Chatsworth, CA, USA) were attached one positioned to the lower back (L5 vertebra) and one the forehead. Prior to the attachment, body hair was removed to ensure optimal sensor adherence, then skin was cleaned with alcohol and dried.

The artificial gravity load, individualized for each participant, was adjusted by increasing the centrifuge's angular velocity. Using integrated software, the ground reaction forces (GRF) at the participants' feet, generated by the centrifuge's rotation, were monitored. Once the desired load, corresponding to 20% of 1RM for squats or 1.5 × body weight for calf raises, was achieved, the experiment commenced. Each participant then performed two sets of 12 repetitions of the squat exercise and subsequently two sets of 12 repetitions of calf raise. Vibrations were activated one second before the first repetition and deactivated one second after the last repetition. Participants were given one minute of rest between sets and exercises. During the rest, the centrifuge was maintained the speed to maintain angular velocity and hence generating AG.

### URVE group

The URVE trials were conducted at the Environmental Physiology and Ergonomics Laboratory in Ljubljana. The URVE exercise was performed with a pendulum device as depicted in Figure 1A. During the exercise, the subjects stood on the vibration platform. Load was administered through the addition and/or removal of weight plates on the side of the pendulum device. Vibration data was recorded by using exactly the same system described in the AGRVE group. Before attaching the accelerometers, the skin was prepared to optimize sensors' placement. The exercise protocol was identical to the one performed by AGRVE participants, as well as the rest between sets and repetitions. To mimic the constant load experienced by participants in AGRVE group, during the rest between exercise, participant in the URVE groups were resting with the load still placed on the shoulders.

### Data analysis

#### Vibration data acquisition

Vibration data was recorded and stored using the same procedure employed as previously reported (43, 44). Data are presented as root mean squared (RMS) values based on 1/3 octave bandwidths (Constant Percentage Bandwidths, CPB) recorded from each sensor. Since this study utilized only a single bandwidth (20 Hz), the data were extrapolated specifically for that range following an evaluation across the entire spectrum. Within DewesoftX (version, Dewesoft d.o.o., Trbovlje, Slovenia), octave analysis utilizing 1/3-octave Constant Percentage Bandwidths (CPB) was conducted for each sensor, sampled at a sampling frequency of 1,000 Hz. The computation of true octave band data, compliant with ANSI S1.11 and IEC 61260 standards, was performed using an analysis window of 33.333 ms (block history), linear averaging, and no frequency weighting. This data was subsequently exported to MS Excel (version, Microsoft Corporation, Redmond, WA, USA), where data from the 20 Hz 1/3-octave band was averaged over the total duration of the exercise. The data was not filtered. Vibration transmission is expressed as the ratio between the values recorded by the sensors. A ratio of 1 indicates complete transmission between the two sensors (i.e., transmission from the foot to the pelvis), while a ratio greater than 1 signifies an amplification of vibrations and a ration below 1 indicates an attenuated transmission.

### Statistical analysis

Vibration data was exported to MS Excel were subsequently analysed with statistical procedures. Given the sample size, normality of the data was assessed using the Shapiro–Wilk test, confirming a normal distribution across all variables. Upon verifying normality, a mixed-effects model was implemented with *exercise* (AGRVE, URVE) and *sensor position* (the body segment where the accelerometer was placed head, pelvis) as fixed factors. Where a significant main effect or interaction between exercise type and sensor position was detected, *post-hoc* pairwise comparisons were conducted using t-tests to identify specific group differences with Bonferroni-Holm method correction for multiple comparisons. The Bonferroni – Holm correction was applied to control the risk of Type I error arising from multiple within-group and between-group comparisons. The correction method was selected for its conservative nature, aimed at minimizing the possibility of incurring on false-positive results. To test vibration transmission, a mixed-effect model was performed with *exercise* (AGRVE, URVE) and *ratio* (the ratios between head/pelvis/foot). Statistical significance was defined *a priori* at *p* < 0.05.

To strengthen the analysis, independent from sample size, a Hedge's G effect size was implemented to quantify the magnitude of possible differences. The choice was done because this works fine with relatively low and unequal sample sizes (45). Statistical analysis and visual representation were conducted with GraphPad Prism 10.0 (Dotmatics, Boston, Massachussets).

## Results

### Squat

**Figure 2:**
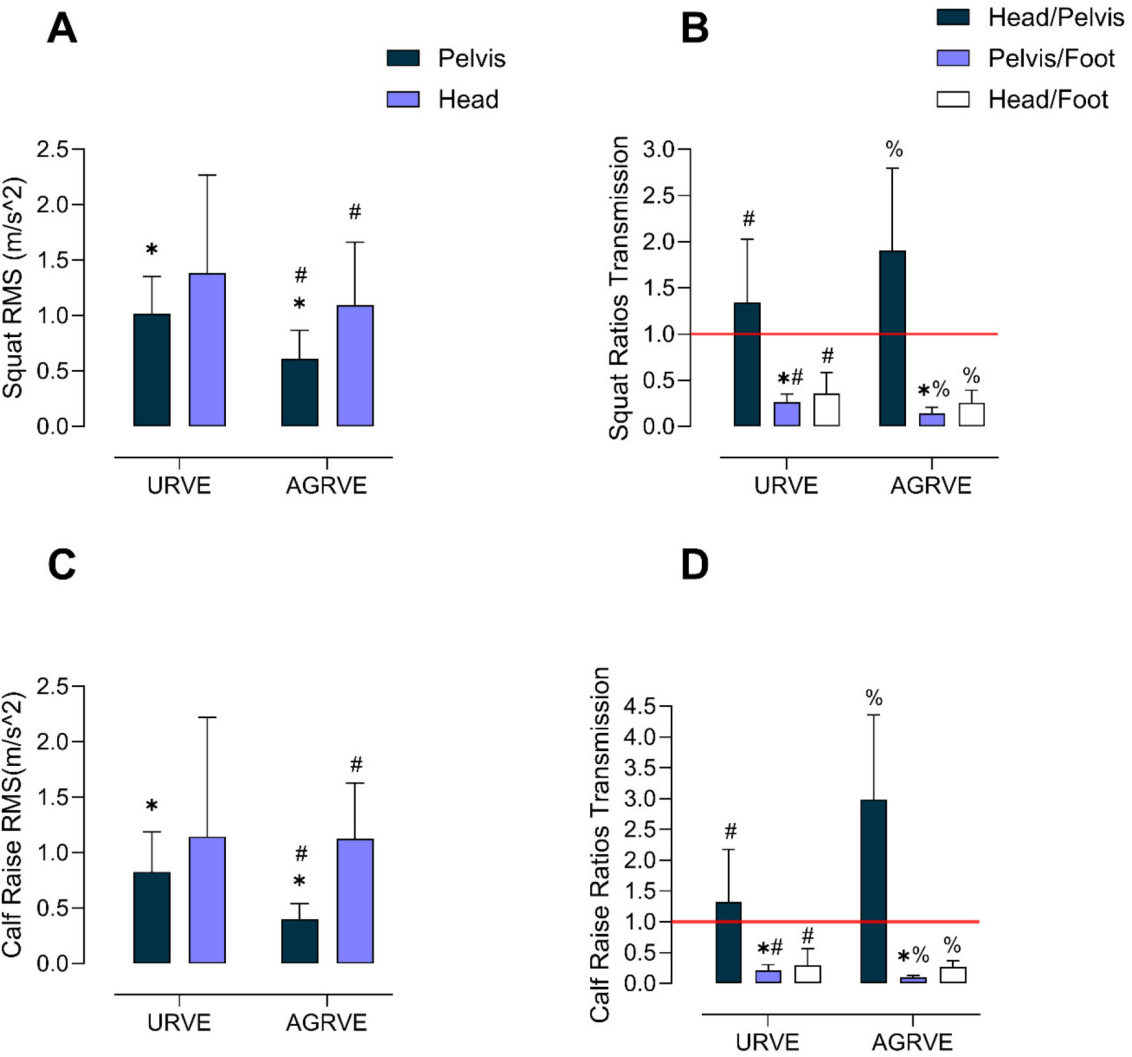
Bar plots illustrating mean ± standard deviation for group comparisons: **(A)** octave band squat RMS, **(B)** squat vibration transmission ratios, **(C)** octave band calf raise RMS, and **(D)** calf raise vibration transmission ratios. Asterisks (*) in panels A and C denote a difference between groups. Hashtag (#) denotes a difference within the same group. Percentage symbols (%) in panels **(B)** and **(D)** a difference within AGRVE and hashtag (#) a difference within URVE group. The horizontal red line in **(B)** and **(D)** marks the threshold for full transmission between two regions: values ≥1 represent full transmission, while values < 1 indicate dampening between regions.

A significant main effect of sensor position was observed [F (1, 13) = 6.49; *p* = 0.02]. Subsequent analysis showed that pelvis RMS values were 51% lower in the AGRVE group compared to the URVE group (*p* = 0.01; G = −1.36) as observed in Figure 2. No significant difference was observed in head RMS values between the AGRVE and URVE groups. No further attenuation of vibrations occurred between the pelvis and head, as no significant differences in RMS values were observed within the URVE group. However, a moderate effect size was noted in URVE (G = 0.54). A statistically significant difference was found within AGRVE (*p* = 0.04, G = 1.10) where head RMS was significantly higher than pelvis RMS.

Vibration transmission ratios analysis confirmed the octave RMS data. A *ratio* effect was recorded [F (2,26) = 50.48; *p* < 0.0001]. Head/Pelvis ratio was statistically significantly higher compared to both Pelvis/Foot and Head/Foot ratios in both groups (*p* < 0.0001) indicating that no further vibration attenuation occurred between the pelvis and head. Instead, an amplification of vibrations was observed, driven by head movement rather than actual vibrations transmitted. In the AG group, this amplification was exaggerated, as noted in previous studies. Between groups, no differences were recorded for the Head/Pelvis ratio, nevertheless a moderate to high effect size was recorded (G = 0.70). The Pelvis/Foot ratio was 44% lower in AGRVE compared to URVE (*p* = 0.008, G = −1.56) meaning a significant reduced transmission between the platform and the pelvis. No statistically significant difference was found for Head/Foot ratio; however, a moderate effect was recorded between groups (G = −0.52).

### Calf raise

As for the squat, vibration transmission was also reduced during calf raises. A *sensor position* effect was recorded [F (1, 13) = 8.56; *p* = 0.01]. AGRVE pelvis RMS values were 52% lower compared to URVE (*p* = 0.01, G = −1.51), while no differences were recorded for head RMS. AS for the squat, no differences were found between pelvis and head RMS within group in URVE, however a statistically significant difference was found within AGRVE (*p* = 0.01, G = 1.97), where head RMS values were higher compared to pelvis as displayed in Figure 2.

For vibration transmission, *exercise x ratio* interaction was recorded [F (2,26) = 9.62; *p* = 0.0007] as well as a main *ratio* effect [F (2,26) = 48.34, *p* < 0.0001] and *exercise* effect [F (1,13) = 5.41, *p* = 0.03]. *Post hoc* comparisons shows that the Head/Pelvis ratio was statistically significantly higher compared to both Pelvis/Foot and Head/Foot in both groups (AGRVE, *p* < 0.0001: URVE, *p* = 0.03). As for the squat, the higher ratio at the head during the calf raise is primarily driven by its actual movement during the exercise rather than the vibration stimulus. In the AGRVE group, this effect is further exaggerated by the action of the SAHC. Between groups, the Head/Pelvis ratio was higher in AGRVE compared to URVE (*p* = 0.02, G = 1.48). The Pelvis/Foot ratio was 58% lower in AGRVE compared to URVE (*p* = 0.007, G = −1.68) confirming the octave RMS recorded and thus meaning in a highly reduced transmission between the vibration platform and the pelvis. No statistically significant difference was found for the Head/Foot ratio. All the main statistical comparisons are presented in Tables 1–4.

**Table 1:** Mean values and *post-hoc* results comparisons between exercise group for root mean square (RMS) values and vibration transmission expressed in ratio during squat and calf raise (CR).

Sensor body position (type of exercise)	URVE (average)	AGRVE (average)	*p*-value
Pelvis RMS (Squat)*	1.02	0.60	*p* = 0.01
Head RMS (Squat)*	1.38	1.09	n.s.
Head/Foot ratio (Squat)	0.33	0.25	n.s.
Head/Pelvis ratio (Squat)	1.27	1.77	n.s.
Pelvis/Foot ratio (Squat)	0.25	0.14	*p* = 0.008
Pelvis RMS (CR)*	0.82	0.40	*p* = 0.01
Head RMS (CR)*	1.13	1.14	n.s.
Head/Foot ratio (CR)	0.28	0.28	n.s.
Head/Pelvis ratio (CR)	1.26	2.77	n.s
Pelvis/Foot ratio (CR)	0.23	0.10	*p* = 0.007

The asterisk indicates that for these values, the unit of measurement is meters per second squared (m/s^2^).

**Table 2:** Comparison of Hedge's G effect size between groups.

Sensor body position	AGRVE – URVE (Squat)	AGRVE – URVE (Calf Raise)
Pelvis	−1.36	−1.51
Head	−0.38	//
Head/Foot ratio	−0.52	//
Head/Pelvis ratio	0.70	1.48
Pelvis/Foot ratio	−1.56	−1.68

Values ≥0.20 or - 0.20 denote a small effect, values ≥0.50 or - 0.50 denote a moderate effect, and values ≥0.80 or - 0.80 denote a large effect.

**Table 3:** *Post-hoc* results comparisons within exercise group for root mean square (RMS) values and vibration transmission expressed in ratio during squat and calf raise (CR).

Exercise group (type of exercise)	Head vs. Pelvis	Head/Pelvis vs. Pelvis/Foot ratios	Head/Pelvis vs. Head/Foot ratios	Pelvis/Foot vs. Head/Foot ratios
URVE (Squat)	n.s.	*p* = 0.02	*p* = 0.0008	n.s.
URVE (Calf raise)	n.s.	*p* = 0.007	*p* = 0.003	n.s.
AGRVE (Squat)	*p* = 0.04	*p* = 0.002	*p* = 0.001	*p* = 0.03
AGRVE (Calf raise)	*p* = 0.006	*p* = 0.001	*p* = 0.001	*p* = 0.04

**Table 4:** Comparison of Hedge's G effect size within groups.

Exercise group (type of exercise)	Head vs. Pelvis	Head/Pelvis vs. Pelvis/Foot ratios	Head/Pelvis vs. Head/Foot ratios	Pelvis/Foot vs. Head/Foot ratios
URVE (Squat)	0.54	2.21	1.93	0.54
URVE (Calf raise)	0.39	1.84	1.64	−0.40
AGRVE (Squat)	1.10	2.97	2.79	−1.12
AGRVE (Calf raise)	1.97	4.01	3.70	2.23

Values ≥0.20 or - 0.20 denote a small effect, values ≥0.50 or - 0.50 denote a moderate effect, and values ≥0.80 or - 0.80 denote a large effect.

## Discussion

### Summary of findings

The findings of this study indicate that vibration transmission is significantly dampened by the lower limbs during exercise, with minimal vibration reaching the spine. Attenuation was greater in the AGRVE group compared to the URVE group, likely due to specific design features of the cradle that further dampened vibrations, body orientation and the potential artificial gravity action. Our findings corroborate previous research suggesting that the lower limbs act as spring-like dampeners of vibration and demonstrate that external additional mechanic cushion can enhance vibration attenuation (46, 47). In this study, both AGRVE and URVE groups performed exercise with partially flexed knees, a position known to amplify the body's natural damping mechanisms (7, 22). In the AGRVE group, the exaggerated flexion observed in participants with the action of the centrifuge (42) likely contributed to the further attenuation of vibrations. This aligns with previous findings that postural adjustments and external factors, such as cushioning, can significantly reduce vibration transmission to the spine and sensitive internal structures.

#### Safety concerns with incorporating vibration in exercise strategies

ISO 2631 and EU directives establish strict limits for vibration exposure, primarily aimed at protecting workers in industrial environments. These standards define thresholds based on vibration frequency, direction, duration, and RMS values to prevent damage such as back pain or nerve disorders. However, these guidelines were not designed for dynamic, exercise-based scenarios like WBV training, where vibrations originate from the feet and are attenuated by the lower limbs before reaching the spine (16, 24). The applicability of such standards remains debatable when vibration experienced during exercise and/or rehabilitation.

In this study, the RMS vibration value during a single 1-minute session was 25.50 m/s^2^, aligning with the ISO exposure limit value (ELV) for 1-minute exposure but exceeding the ELV for 30 min, which is set at 4.60 m/s^2^. Adhering strictly to ISO standards would limit WBV training durations to less than 1 min, insufficient for eliciting meaningful musculoskeletal adaptations. This discrepancy highlights the limitations of applying industrial safety standards to exercise contexts, particularly when WBV is used as a countermeasure for musculoskeletal health. As an example, the ground reaction forces (GRFs) and vibrations experienced during skiing may exceed the occupational exposure limits specified by the ISO standard (43, 44). Recreational skiers may be exposed to vibrations exceeding ISO standard limits for several hours a day without any negative consequences of the vibration exposure. The exceptions may be professional skiers, either competitive athlete and ski instructors (43, 44), who are exposed to the vibrations on a daily basis and in some cases throughout the year.

The harmful effects of vibrations are linked to the tolerance of the vertebral column and surrounding internal organs to vibration. Some studies suggest that exceeding ISO limits during WBV training does not result in immediate harm (1, 16, 48), others report increased risks of back pain and lumbar disorders when exposure exceeds these thresholds (49, 50). For example, studies on skiers have shown that high ground reaction forces combined with vibration exposure significantly increases the risk of low back pain (44) A review from Griffin (51), critically pointed out how scientific evidence is not sufficient to clearly define applicable dose-response relationship between WBV and back or other disorders. It has been observed that even tough ISO standards might overestimate the vibration dose value which might lead to health issues, it is important to mention that in epidemiological studies following specific criteria to meet ISO standards, there was evidence of increased back pain, nerve pain and lumbar intervertebral disc disorders (49). This conclusion is also supported by field studies on skiers (43, 50), where vibrations exposure values were compared to EU directive.

#### Implications for astronaut training during space missions

WBV training for astronauts represents a unique challenge. It is currently being investigated as a potential exercise countermeasure, in combination with artificial gravity, for astronauts during deep space missions. Unlike typical WBV sessions, which are brief and infrequent, astronauts may engage in daily training regimens during extended missions, such as those to Mars. Astronauts undergo daily training regime for several hours and hence integrating vibration into these routines would significantly increase the cumulative vibration exposure, especially when considering the extended durations required for future Mars missions (25). Nonetheless, in this study resistance vibration exercise performed on a centrifuge, generated less RMS at pelvis compared to upright exercise, so this type of exercise might be carefully considered as a possible exercise countermeasure to space exploration. Currently, existing ISO standards are not directly applicable to the context of daily vibration exposure for future astronaut training protocols. Therefore, future studies investigating AGRVE should consistently include the assessment of vibration transmission. This approach would facilitate the establishment of safety thresholds beyond which exposure to such exercise modalities may pose health risks to astronauts and, more broadly, to members of the general population who may adopt this novel training method.

#### Vibration transmission

Vibration transmission is a critical factor to consider during physical activities. Several studies have investigated upright whole-body vibration transmission either with (18) or without exercise (7, 22). It is evident that the majority of the acceleration provided by the vibration platforms is dampened in the lower limbs, resulting in negligible vibrations reaching sensitive areas such as the lower back.

The results of this study align with previous research, showing that vibrations were significantly dampened by the lower limbs in both groups. As demonstrated by Rubin and colleagues (47), foot to pelvis ratio is reduced to 0.5 for frequencies below 20 Hz and even to 0.25 for frequencies equal or above 20 Hz, which is consistent with the observations in the present study. In the present study, given the equipment, both groups were performing both exercise with knees partially flexed. This was exaggerated in the SAHC as reported previously (42). Namely, the movement in the centrifuge is somehow constrained in naïve users of the device. This might further explain why vibration transmission in more dampened in AGRVE compared to URVE.

Pollock et al. (7) reported no further reduction between lower back/pelvis and head, indicating that legs are the main structure dampening vibrations. The exact mechanisms of this phenomenon are unclear. In the present study, during upright exercise, the effect size analysis shows that head RMS is higher than pelvis. In AGRVE this was confirmed also by the analysis of variance. This difference is explained by the fact that the head had greater freedom of movement compared to pelvis and the RMS are more related to the actual movement during exercise than vibration, as shown also during field studies conducted on skiers (44). Hence suggesting no potential risk of head damage due to vibrations. Previous research (16) has proposed the concept of human body and its segments, as a spring-like system. Calf and thigh muscles are usually referred to as spring-dashpot systems, which can store and absorb energy in muscle and ligaments, hence reducing the vibratory stimulus travelling through the body. Vibration transmission is affected by many factors, including physical characteristics (e.g., bone, soft tissue, muscular activity, posture, etc.) and external factors (e.g., types of shoes, device used, etc.) (6, 7, 21, 22, 46). Previous research (7, 22) has demonstrated that the reduced transmission above the knee level at frequencies ≥15 Hz may be attributed to the active damping, wherein muscles contract to mitigate adverse effects. The reduced transmission ratios in AGRVE compared to URVE can be attributed to the configuration of the cradle used during the exercises. Participants were positioned on their backs during centrifugation, with accelerometers placed at the L5 vertebra. To minimize noise and pressure on the sensor, the board on which participants lay was equipped with two foam cushions running along its length, separated by a vertical space. The *pelvis* accelerometer was placed within this space to prevent it from being compressed by the lower back, thereby avoiding altered results. This cushioning likely partially contributed to the lower transmission and RMS values observed in AGRVE compared to URVE by absorbing part of the vibrations before they reached L5 aligning with previous research indicating the further dampening effect of external factors (46).

### AGRVE vs. URVE

The reduced root mean square (RMS) values and vibration transmission observed during AGRVE may be attributed to several interacting factors. As mentioned in previous section, participants in the AGRVE condition were positioned horizontally on a custom-designed cradle. Although the accelerometers were not in direct frictional contact with the cradle, the interface between the participant's body and the cushioning material may have contributed to attenuating vibration transmission compared to the URVE, where contact is limited to the feet. Furthermore, the horizontal posture alters body weight distribution and increases surface contact area, potentially enhancing the absorption and dissipation of vibratory energy through the supporting structure. Additionally, AGRVE is characterized by a gravitational gradient that increases from the head to the feet, resulting in elevated compressive forces acting on the lower extremities. This mechanical loading likely increases musculoskeletal stiffness, thereby facilitating greater damping of vibration signals. This interpretation is supported by previous findings from studies on upright WBV, where higher muscular activation and segmental stiffness, ultimately lead to a reduction in vibration transmission throughout the body (16, 21).

### Limitations

The results of this study should be interpreted in light of several considerations. First, a light external load was utilized, selected to replicate a training-like effort while avoiding excessive fatigue that could compromise exercise technique and, consequently, the accuracy of vibration data collection. Future research should explore varying load intensities during artificial gravity exposure to determine whether increased muscular effort further attenuates vibration transmission. Additionally, this study employed a single vibration frequency, as previously described. Subsequent investigations should incorporate multiple frequencies in conjunction with artificial gravity to examine the corresponding vibration behaviour. It is important to note that at higher vibration frequencies, there is an elevated risk of foot detachment and floating over the platform, potentially increasing injury risk during complex exercise such as AGRVE. Lastly, while the sample size used aligns with prior studies on vibration transmission, future research should aim to include a larger cohort to enhance the statistical power and generalizability of the findings.

## Conclusion

Vibration transmission was significantly attenuated in both AGRVE and URVE groups, with greater protection observed in AGRVE. While WBV offers potential benefits as a training stimulus, the vibration intensities recorded in this study exceeded ISO and EU limits for safe exposure, potentially raising concerns about its prolonged use in astronaut training regimens. For extended missions, such as those to Mars, further research is needed to balance the anabolic benefits of WBV with its potential risks, considering cumulative exposure over time and its implications for musculoskeletal health. Future research should aim to investigate the effects of prolonged exposure to AGRVE on musculoskeletal adaptations, vestibular function, and neuromuscular coordination. Such investigations are essential to determine whether AGRVE elicits beneficial physiological responses that may surpass those induced by traditional exercise modalities, while also addressing potential concerns related to the cumulative effects of daily vibration exposure.

## Data Availability

The raw data supporting the conclusions of this article will be made available by the authors, without undue reservation.
